# *DRAM2* acts as an oncogene in non-small cell lung cancer and suppresses the expression of p53

**DOI:** 10.1186/s13046-019-1068-4

**Published:** 2019-02-12

**Authors:** Muli Wudu, Hongjiu Ren, Linping Hui, Jun Jiang, Siyang Zhang, Yitong Xu, Qiongzi Wang, Hongbo Su, Xizi Jiang, Runa Dao, Xueshan Qiu

**Affiliations:** 1 Department of Pathology, First Affiliated Hospital and College of Basic Medical Sciences, China Medical University, Shenyang, China; 2 Department of Pathology, Fourth Affiliated Hospital of China Medical University, Shenyang, Liaoning China; 3 Center of Laboratory Technology and Experimental Medicine, China Medical University, Shenyang, Liaoning China

**Keywords:** Non-small-cell lung cancer, *DRAM2*, Cell migration, Cell proliferation, P53, P21

## Abstract

**Background:**

Damage-regulated autophagy modulator 2*(DRAM2*) is associated with autophagy processes. However, the role of *DRAM2* in the progression of human neoplasms is still unknown. Here, we show that *DRAM2* may act as an oncogenic regulator in non-small cell lung cancer (NSCLC).

**Methods:**

Tumor specimens from 259 NSCLC patients were collected and analyzed. Transwell migration, cell cycle analysis, MTT and colony formation assays were performed to determine the effect of *DRAM2* overexpression and knockdown on NSCLC-cell migration and proliferation. Western blotting confirmed the expression of DRAM2, p53, and the other involved proteins.

**Results:**

*DRAM2* was preferentially upregulated in NSCLC tissues and higher expression of *DRAM2* in NSCLC correlated with tumor node metastases stage and lymph node metastasis. Additionally, *DRAM2* overexpression promoted cell metastasis and proliferation in vitro, while knockdown of *DRAM2* expression yielded opposite result. Furthermore, *DRAM2* overexpression increased the expression of proteins RAC1, RHOA, RHOC, ROCK1, and decreased RHOB expression, all of which are cell migration factors. *DRAM2* overexpression also increased proteins CDK4, CyclinD3, and decreased p27 expression, all of which are cell cycle-related factors. Consistently knocked down *DRAM2* had the opposite effect. We also found that *DRAM2* expression was negatively correlated to p53 expression. Knockdown of *DRAM2* caused an increase of p53 and p21 expression, and overexpression of p53 caused a decrease of DRAM2 expression. Finally, absence of p53 did not influence the function of *DRAM2* in NSCLC, but overexpression of p53 repressed its function.

**Conclusions:**

*DRAM2* plays an oncogenic role in NSCLC via regulating p53 expression. Therefore, *DRAM2* may act as an oncogene in NSCLC and could serve as a prognostic factor and potential target for NSCLC treatment.

## Background

The gene, *damage-regulated autophagy modulator 2* (*DRAM2*), also known as *TMEM77*, is located on chromosome 1p13.3 and encodes a six putative transmembrane domain-containing protein of 176 amino acids [1]. Mutations in this gene are associated with retinal dystrophy [2, 3]. MicroRNA 125b-1, microRNA 144, and lncRNA HOTAIRM1 can bind to the mRNA of this gene and produce a disease state [4–6]. *DRAM2* shares significant homology with *DRAM*, displaying a 36.7% similarity in amino acids and both are co-localized in lysosomes [7]. Although *DRAM* is one of the important regulatory factors of p53-mediated autophagy [8], the relationship between *DRAM2* and p53 remains controversial. Some researchers have suggested that unlike *DRAM*, *DRAM2* is not involved in p53 and autophagy [9], while other publications claim *DRAM2* is involved in p53-induced cell death,and promotes the autophagy process [7]. Given that the relationship of *DRAM2* with p53 remains a topic of debate, exploration of this association is needed.

With the exception of serving as an autophagy-related protein in a few types of tumors [5, 7, 10–12], the protein DRAM2 had not been thoroughly studied in the context of cancer. Some researchers posit that genes encoding transmembrane or secretory proteins, which are expressed specifically in cancers, may serve as ideal biomarkers for cancer diagnosis, and if the gene production is involved in the neoplastic process, the gene may become a therapeutic target [13]. Based on this, the transmembrane gene, *DRAM2*, was chosen as a potential therapeutic target and found to be expressed at much higher levels in gastric cancer than in normal tissues,however no further research has been performed [14]. Given these results, *DRAM2* expression and the role it plays in other cancers is worth investigating.

Lung cancer is the most frequently diagnosed malignancy resulting in the highest mortality rates among all cancers [15, 16]. Approximately 85% of lung cancer patients are diagnosed with non-small cell lung cancer (NSCLC) [17] and the 5-year survival rate of NSCLC remains very low [18]. Meanwhile, the tumor suppressor, p53, which is mutated in almost 50% of tumors [19], plays an important role in oncogenic signaling [20–23]. Thus, in this study, we aimed to elucidate the expression and function of *DRAM2* in the progression of NSCLC and the relationship between *DRAM2* and p53 in NSCLC, which may provide valuable insights into the regulatory mechanism of lung cancer and a novel therapeutic target.

## Methods

### Patients and specimens

Our research was approved by the Medical Research Ethics Committee of China Medical University and informed consent was obtained from all patients.Specimens of 259 non-small cell lung cancer patients were randomly obtained from the Pathology Archive of the First Affiliated Hospital of China Medical University from 2014 to 2017. All enrolled patients underwent curative surgical resection without having prior chemotherapy or radiation therapy.

### Immunohistochemical method and result analysis

The paraffin-embedded NSCLC tissue was collected and sliced into 4 μm sections. The sections were deparaffinized in xylene, rehydrated in a graded alcohol series, and treated with 0.01 mol/L citrate buffer (Maixin-Bio, Shenzhen, China) under high pressure for 2 min to repair heat antigens. Endogenous peroxidase activity was blocked by H_2_O_2_ (0.3%), and the sections were incubated with goat serum (Maixin-Bio, China) at 37 °C for 20 min to reduce non-specific binding. Next, the sections were incubated with anti-DRAM2 rabbit polyclonal antibodies (1200 dilution; Abcam, Cambridge, UK) at 4 °C for 18 h, and the reaction was visualized via immunohistochemical staining by the Elivision super HRP (Mouse/Rabbit) IHC Kit (Maixin-Bio, China) and 3,3′-diaminobenzidine (DAB) color developing, and redyeing with hematoxylin. Known positive slices of NSCLC were used as the positive control and phosphate buffered saline (PBS) replaced the primary antibody as the negative control. The intensity of DRAM2 staining was scored as follows: 0 (no staining), 1 (weak staining), 2 (moderate staining), and 3 (strong staining). Percentage scores were assigned as follows: 1 (0–25%), 2 (26–50%), 3 (51–75%), and 4 (71–100%). The scores of each tumor sample were multiplied to give a final score ranging from 0 to 12, normal bronchia was scored as well. Tumor samples with scores ≥4 were defined as DRAM2 overexpression, and those with scores < 4 were categorized as showing weak or negative expression.Choosing 4 as the critical value was because the score of DRAM2 over expression in bronchus was ≥4 and the score of low expression in bronchus was < 4.

### Cell culture and treatment

The lung cancer cell lines A549, H1299, H460, H292, H661, and SK-MES-1 were purchased from the Cell Bank of the China Academy of Sciences (Shanghai, China), and normal bronchial epithelial HBE cells were obtained from ATCC (Manassas, VA, USA). A549, H292, H1299, H460, and H661 cells were cultured with Roswell Park Memorial Institute 1640 medium (Gibco, Waltham, MA, USA). SK-MES-1 cells were cultured in minimal essential medium (Gibco), and HBE cells were cultured in DMEM (Gibco). All media were supplemented with 10% fetal bovine serum (FBS, CLARK, South America). The cell lines we used were all p53 non-mutant cells.The cells were maintained in a 5% CO_2_ incubator at 37 °C.

Cell transfection was carried out using Lipofectamine 3000 reagent (Invitrogen, Waltham, MA, USA) according to the manufacturer’s instructions. In *DRAM2* knockdown experiments, cells were transfected with *DRAM2*-specific small interfering RNA (siRNA) and negative control (NC) siRNA (Ruibo, Guangzhou, China) for 48 h,and the siRNA sequences for DRAM2 was GGACTGATTTAGAACAGAA.For *DRAM2* overexpression, cells were transfected with a *DRAM2* expression plasmid, TP53 expression plasmid, and the corresponding empty pcmv6 vector, which were all purchased from Origene (Rockville, MD, USA).

### Immunofluorescence

Lung cancer cells cultured on coverslips in 24-well plates for 24 h were fixed in 4% paraformaldehyde for 15 min and permeabilized with 0.1% Triton X-100 for 10 min, washed three times with PBS, followed by blocking in BSA (5%) for 2 h at 25 °C, and incubation with the anti-DRAM2 antibodies (1:200; Abcam) overnight at 4 °C. Next, cells were washed and incubated for 2 h at 25 °C with a tetramethylrhodamine (TRITC)-conjugated secondary antibody and then washed. Cells were counterstained with 4,6-diamino-2-phenyl indole (DAPI) for 10 min at 25 °C. Cell images were captured using an Olympus FV1000 laser-scanning confocal microscope (Olympus, Tokyo, Japan).

### RNA extraction and real-time PCR

Total RNA was isolated using Trizol (Invitrogen, NY, USA) following the manufacturer’s protocol. The concentrations of RNA samples were measured with spectrophotometers. Quantitative real-time (RT)-PCR was carried out in a 7900HT Fast RT-PCR System (Applied Biosystems, Foster City, CA, USA) using SYBR Green RT-PCR master mix (Takala, Dalian, China) in a total volume of 20 μL under the following cycling conditions: 95 °C for 30 s then 45 cycles of 95 °C for 5 s and 60 °C for 30 s. A dissociation step was performed to generate a melting curve to confirm the specificity of the amplification. *β-actin* was used as the reference gene. The relative levels of gene expression were represented as Δ*C*t = *C*t gene − *C*t reference, and the fold change of the gene expression was calculated by the 2 − ∆∆*C*t method [24]. The experiments were repeated in triplicate. The primer sequences were as follows:

*DRAM2* reverse, 5’-ATGTAAGTGGAGCTGTGCTTACCTTTGGT-3′;

*DRAM2* forward, 5’-ACTGTGCAAAACTGAGCAAGTCAG-3′;

*β-actin* reverse,5′- ATGTACCCTGGCATTGCCGA-3′;

*β-actin* forward,5′- ACACGGAGTACTTGCGCTCA-3′.

### Western blot analysis

Proteins directly influenced by cell migration and those involved in cell proliferation and cell cycle progression, were analyzed by western blotting. Total protein from cells and tumor tissues was extracted in lysis buffer (P0013; Beyotime Biosciences, Shanghai, China) and a protease-inhibitor cocktail(B14002; Biotool, Shanghai, China) and/or a phosphatase-inhibitor cocktail (B15002; Biotool), according to manufacturer instructions and quantified using the Bradford method [25]. Proteins (80 μg/lane) were separated by 10% sodium dodecyl sulfate, sodium salt-polyacrylamide gel electrophoresis (SDS-PAGE) gels, transferred onto polyvinylidene fluoride (PVDF) membranes (Millipore, Germany), which was then exposed to 5% non-fat dried milk (232,100; Becton Dickenson, Franklin Lakes, NJ, USA) in tris-HCl buffer solution (TBS)-Tween buffer for 2 h at 25 °C before incubation overnight at 4 °C with primary antibodies (Table 1). The PVDF membrane were then washed three times before incubation for 2 h at room temperature with horseradish peroxidase (HRP)-conjugated goat antibodies to rabbit / mouse IgG. Immune reactivity was detected by electrochemiluminescence (ECL) (Thermo Fisher Scientific, Waltham, MA, USA) using a BioImaging System (UVP Inc., Upland, CA, USA). Relative protein expression was calculated after normalization to glyceraldehyde 3-phosphate dehydrogenase (GAPDH) used as a loading control.

**Table 1:** list of antibodies used for western blot

Antibody name	Brand	Catalog number	Host	Dilution
DRAM2	Abcam	121,554	Rabbit	1:200
DRAM2	SIGMA	hpa-18,036	Mouse	1:1000
GAPDH	Beyotime	AF0006	Mouse	1:1000
RAC1	Proteintech	66,122-I-lg	Mouse	1:500
RhoA	Cell SignalingTechnology Inc.	2117	Rabbit	1:1000
RhoB	Santa Cru.	sc-180	Rabbit	1:100
RhoC	Cell SignalingTechnology Inc.	3430	Rabbit	1:1000
Rock1	Wanlei Bio.	Wl01761	Rabbit	1:500
CDK4	Cell SignalingTechnology Inc.	12,790	Rabbit	1:1000
CyclinD3	Cell SignalingTechnology Inc.	2936	Rabbit	1:1000
p27	Proteintech	25,614-I-AP	Rabbit	1:500
P53	Proteintech	10,442-I-AP	Rabbit	1:500
P21	Proteintech	10,355-I-AP	Rabbit	1:500

**Table 2:** Correlation between DRAM2 expressiom and clinicopathological factors in 259 NSCLC patients

Clinicopathological factors	Number of patient	DRAM2 negative or weak expression	DRAM2 over expression	P
Gender				
Male	163	59	104	0.498
Female	96	30	66	
Age				
> 60	121	41	80	0.896
≤ 60	138	48	90	
Histology				
Squamous cell carcinoma	103	37	66	0.69
Adenocarcinoma	156	52	104	
Differentiation				
Well	118	43	75	0.599
Moderate-Poor	141	46	95	
Lymph node metastasis				
Negative	148	59	89	**0.035**
Positive	111	30	81	
TNM stage				
I	114	48	66	**0.025**
II + III	145	41	104	

*p < 0.05; **p < 0.01

### Cell migration analysis

Cell migration assays were carried out in 24-well Transwell chambers containing inserts with a pore size of 8 μm (Costar, Washington, DC, USA). Cells were trypsinized after 24 h transfection, and 8 × 10^4^ cells were transferred to the upper Transwell chamber in 100 μL medium supplemented with 2% FBS, with 600 μL medium supplemented with 10% FBS in the lower chamber. After incubation for 24 h, cells on the upper membrane surface were removed with a cotton tip, and those that passed through the membrane were fixed with polyformaldehyde and stained with hematoxylin. The number of migrated/invaded cells was counted in 10 randomly selected fields under a microscope at 200× magnification. All experiments were repeated independently at least three times under identical conditions.

### Cell proliferation assay

Cells were seeded in five 96-well plates at a concentration of 3000 cells/100 μL per well and evaluated daily for 5 d. Every day, we chose one of the five plates and added media containing 3-(4, 5-dimethylthylthiazol-2yl-)-2, 5-diphenyl tetrazolium bromide (MTT) (10 μL/well), and the remaining plates were cultured in a 5% CO_2_ incubator at 37 °C. The plate with MTT was incubated at 37 °C for 4 h, then the supernatant was removed, and 150 μl dimethyl sulfoxide (DMSO) was added to dissolve the crystals. Absorbance was measured at 490 nm using a microplate reader.

### Cell cycle assay

The cells were harvested by trypsin digestion 48 h after transfection and washed in cold PBS before fixation in cold 75% alcohol at 4 °C overnight. Alcohol was removed and cells were again washed with cold PBS. Cells were stained with propidium iodide solution (KeyGEN BioTECH, China) containing 20 μg/ml RNase, and incubated at room temperature for 30 min. A FACS Calibur (BD,USA) flow cytometer was used to analyze the cell population. After filtration by a nylon mesh filter, cell cycle analysis was performed on a fluorescence-activated cell sorter (FACS, FACSVerse). Data were analyzed using FlowJo software (Version 7.6.1, Tree Star Software, San Carlos, CA, USA).

### Colony formation assays

Cells were seeded in 6-wells plates at a density of 500 cells/well and allowed to grow undisturbed for 10–15 d at 5% CO_2_, 37 °C. The cells were then washed with PBS, fixed in 4% paraformaldehyde for 15 min and stained with hematoxylin. Colonies with more than 50 cells were counted. At least three independent experiments were carried out under identical conditions.

### Statistical analysis

SPSS 16.0 software (SPSS Inc., Chicago, IL, USA) was used for data analysis. Correlations between *DRAM2* expression and clinicopathological features were examined by chi-squared test, and differences between cell groups were compared by paired *t*-test. Two-sided *p*-values < 0.05 were considered statistically significant. *p < 0.05, **p < 0.01, ***p < 0.001.

## Results

### DRAM2 was overexpressed in NSCLC tissues and has clinical significance

**Fig. 1:**
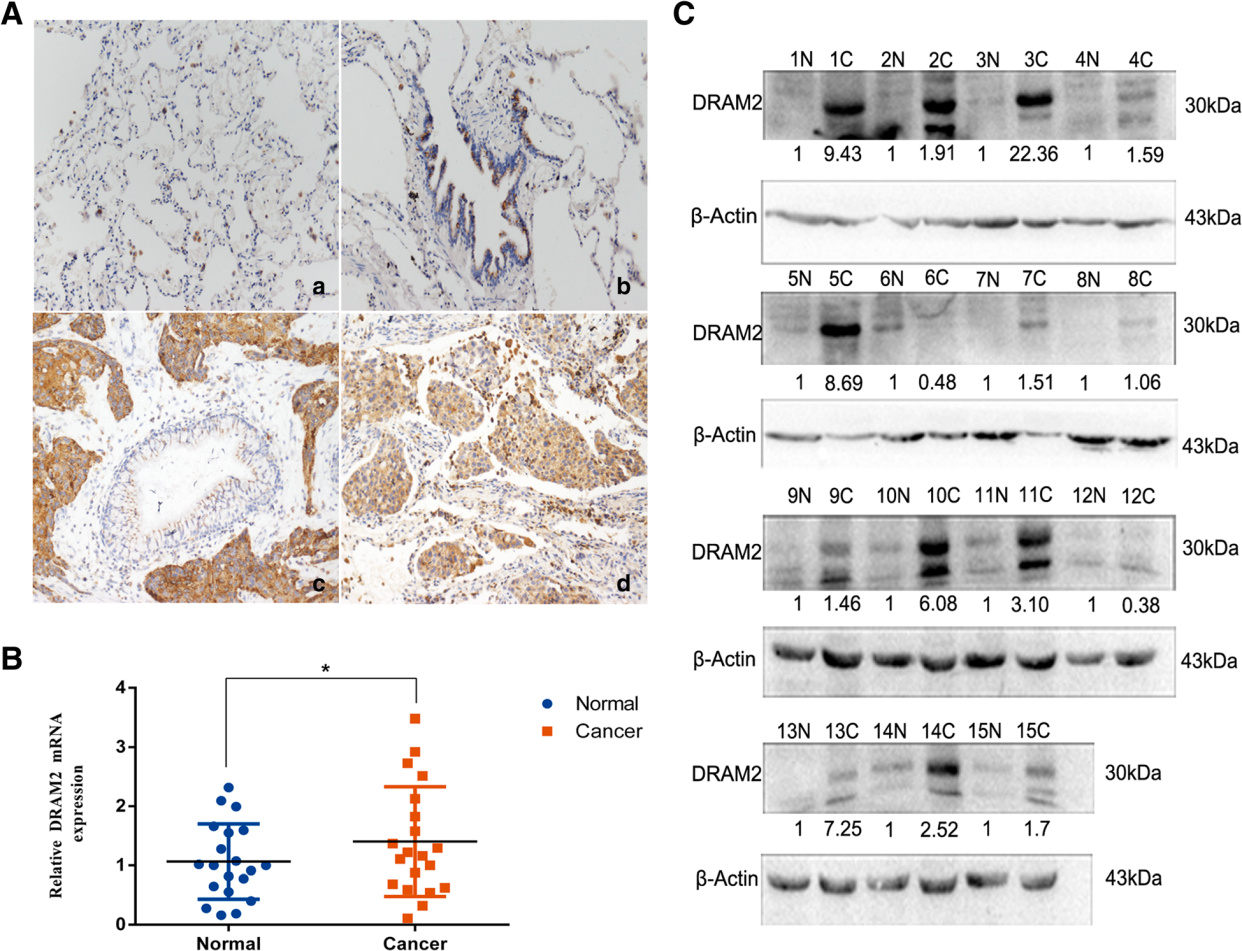
Expression of DRAM2 in non-small cell lung cancer (NSCLC). **a**. *DRAM2* protein expression analyzed by immunohistochemistry in (*a*) alveolar and (*b*) normal bronchial epithelial cells; (*c*) squamous cell carcinoma and (*d*) adenocarcinoma. Magnification, × 200. **b**. Relative expression of *DRAM2* mRNA in 20 pairs of NSCLC samples and adjacent normal lung tissues.*DRAM2* overexpressed in NSCLC samples and have statistical significance,*p* = 0.0466. **c**. Western blot showing the detection of *DRAM2* and p53 in the lysates of 15 paired tumor (C) and adjacent normal tissues (N). β-actin was used as a loading control. The results were quantified using ImageLab, and intensity values were normalized to the β-actin band.The relative expression was quantified below

To explore the expression and subcellular localization of *DRAM2* in NSCLC, we analyzed 259 cases of NSCLC and adjacent noncancerous clinical tissue specimens. Immunohistochemical staining showed that *DRAM2* was localized to the cytoplasm (Fig. 1a). According to established principles for evaluating immunostaining, the expression of *DRAM2* was negative in the normal alveolar (Fig. 1A-a), and negative or very weakly positive in the normal bronchi (Fig. 1A-b), but relatively higher in squamous carcinoma tissues (Fig. 1A-c) and adenomatous carcinoma tissues (Fig. 1A-d).

Next, to investigate whether *DRAM2* expression was associated with the progression of NSCLC, we analyzed the correlation of *DRAM2* expression with the clinicopathological characteristics of the lung cancer patients based on the immunohistochemical staining data. As shown in Table 2, *DRAM2* overexpression was associated with tumor node metastases (TNM) stages (*p* = 0.025) and lymph node metastasis (*p* = 0.035), but did not correlate with age, biological sex, histology, or tumor differentiation (*p* > 0.05).

To confirm *DRAM2* was overexpressed in tumor tissue, we analyzed *DRAM2* mRNA expression in 20 pairs of fresh NSCLC samples via RT-PCR (Fig. 1c) and 15 pairs of tissue specimens, which corresponded to RT-PCR to analyze *DRAM2* expression through western blotting (Fig. 1d), and obtained the same results. Collectively, these observations indicate that the *DRAM2* was overexpressed in NSCLC compared with the adjacent normal lung tissues, and its overexpression was associated with TNM stages and lymph node metastasis of NSCLC patients.

### DRAM2 is located in cytoplasm and highly expressed in NSCLC cell lines

**Fig. 2:**
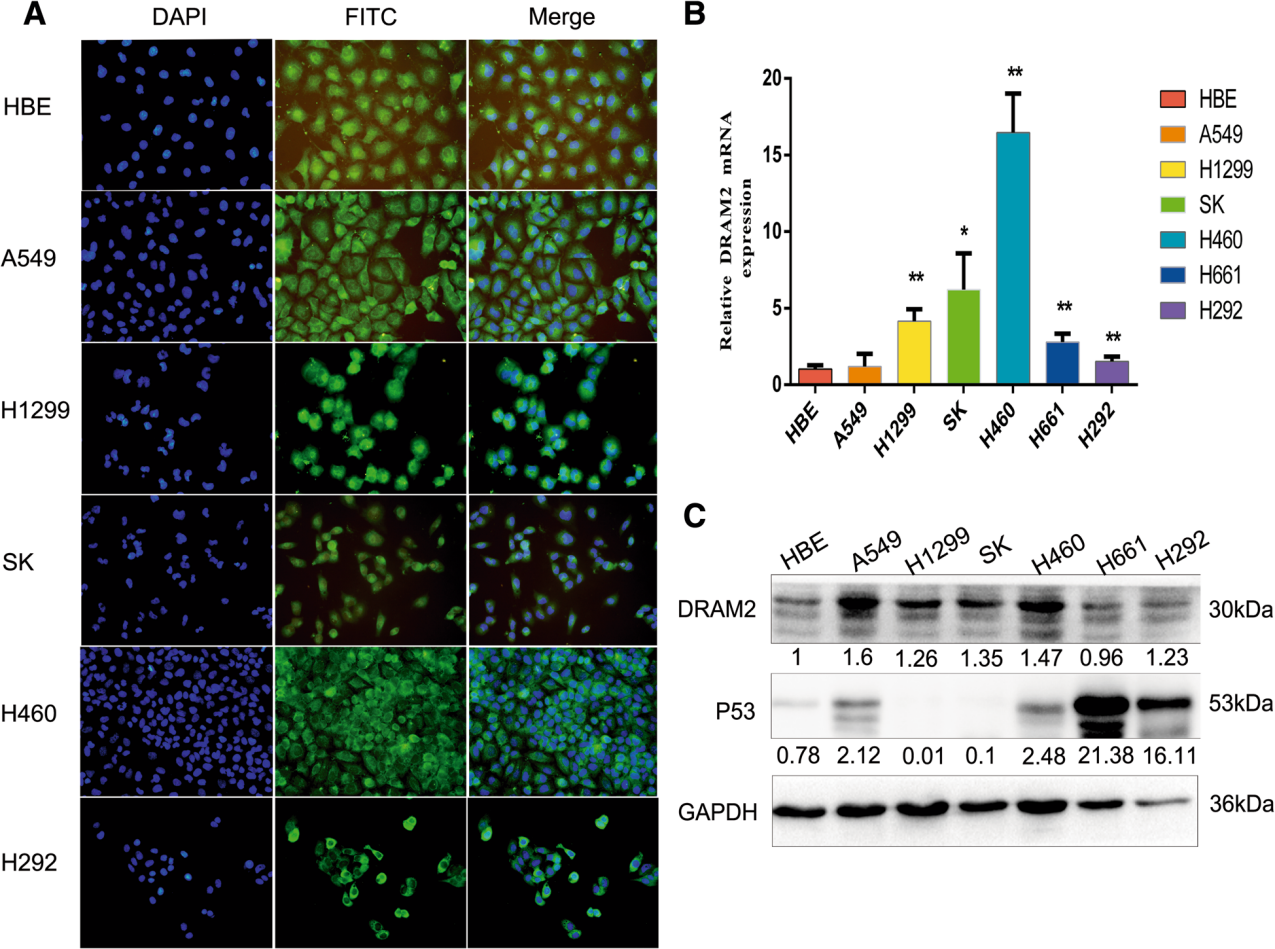
DRAM2 sub-location and expression in non-small cell lung cancer (NSCLC) cell lines. **a**. Immunofluorescence assays were performed to detect *DRAM2* sub-locations in NSCLC cell lines. *DRAM2* was located in the cytoplasm surrounding around nucleus. **b**. Relative expression of *DRAM2* mRNA in six lung cancer cell lines and a normal bronchial cell line (HBE).Compared the expression of *DRAM2* in those cell lines with HBE,A549 got no statistical significance,H1299 *p* = 0.0093,SK *p* = 0.049,H460 *p* = 0.0072,H661 *p* = 0.009,H292 *p* = 0.0055.*p < 0.05, **p < 0.01, ***p < 0.001. **c**. *DRAM2* protein levels in NSCLC cell lines assessed by immunoblotting and analyzed with ImageLab software.The relative expression was quantified below

The expression of DRAM2 and its subcellular distribution in six NSCLC cell lines (A549, H1299, SK-MEM-1, H460, H661, and H292) and a normal bronchial epithelial cell line (HBE) was evaluated usingimmunofluorescence analyses, RT-PCR, and western blot, respectively. We found that *DRAM2* localized to cytoplasm, surrounding the nucleus (Fig. 2a). both by RT-PCR (Fig. 2b) and western blotting (Fig. 2c). Comparing the expression of DRAM2 in NSCLC cell lines with HBE, we determined that DRAM2 was overexpressed in these NSCLC cell lines. The lowest expression was found in HBE in RT-PCR and *p* value was calculated for each cell line(Fig. 2b). Western blotting showed the lowest expression was found in H661 and the highest expression was found in A549 (Fig. 2c).

### DRAM2 promotes migration of NSCLC cells

**Fig. 3:**
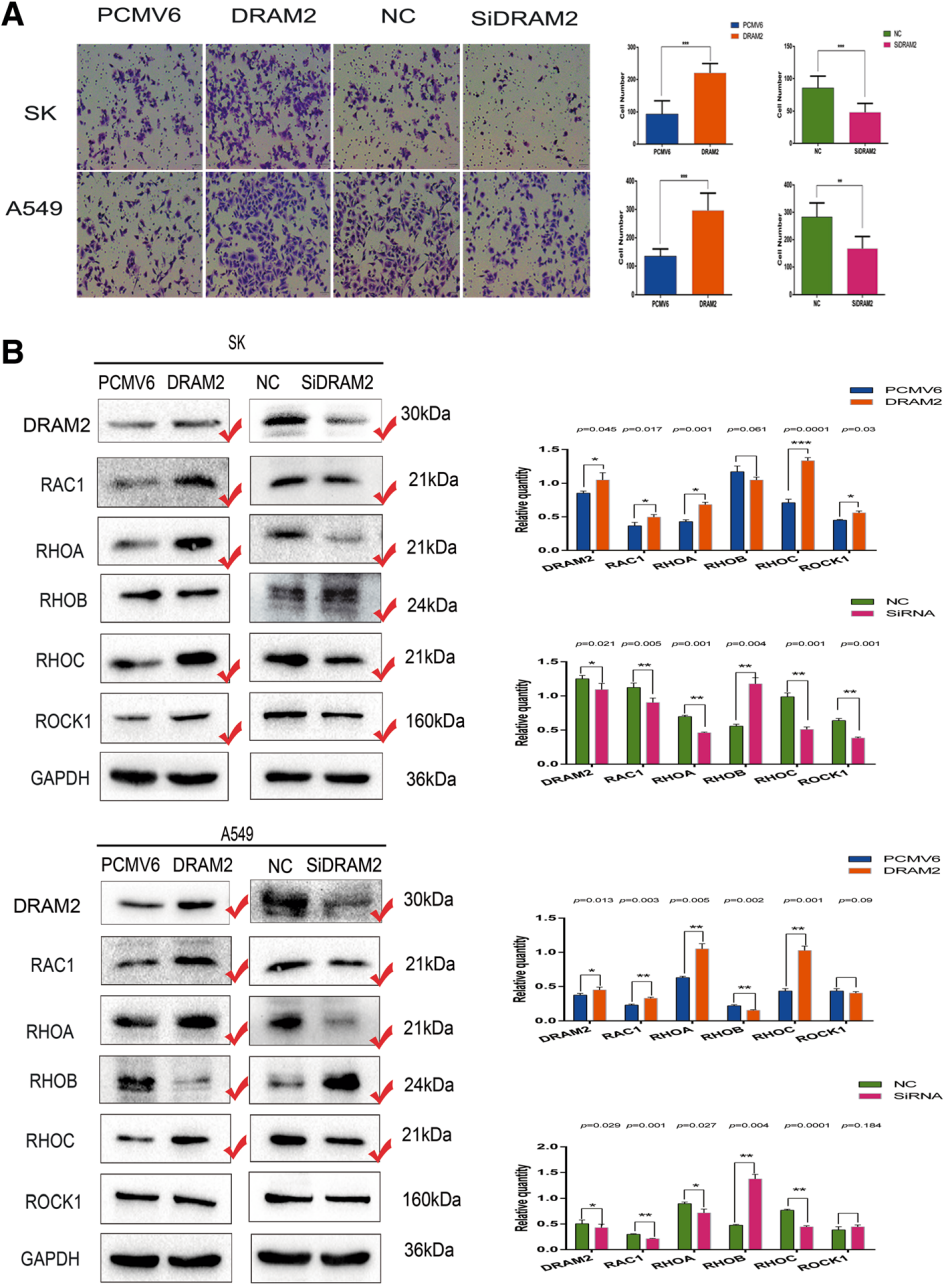
Expression of *DRAM2* affected the migration of non-small cell lung cancer (NSCLC) cells. **a**. Transwell migration assays were performed to analyze cell migration in the context of *DRAM2* overexpression and downregulation in SK and A549 cells, respectively. Cells that migrated to the lower chamber were stained with hematoxylin and counted. *DRAM2* overexpression enhanced cell migration and *DRAM2* knockdown inhibited cell migration. *p*-values for SK were: *DRAM*2 vs PCMV6, *p* < 0.0001; Si*DRAM2* vs negative control siRNA, *p* = 0.0002. *p*-values for A549 were: *DRAM2* vs PCMV6, *p* = 0.0001; Si*DRAM2* vs NC, *p* = 0.0019. *p < 0.05, **p < 0.01, ***p < 0.001. **b**. Effects of DRAM2 levels on the expression of proteins associated with cell migration in transfected SK and A549 cells. The gray level of the proteins were detected, the corresponding *p* value were provided at the top of the graphs and those changed proteins were marked.*p < 0.05, **p < 0.01, ***p < 0.001

Considering the expression of *DRAM2* did not correlate with histology (Table 2), we chose to upregulate and silence *DRAM2* expression in A549 (adenocarcinoma) and SK-MEM-1 (squamous carcinoma, hereinafter referred to as SK) for subsequent experiments. Based on the result that *DRAM2* correlated with lymph node metastasis, we first explored the effects of *DRAM2* on cell migration of NSCLC, via transwell migration experiment, and the difference in transwell migration was statistically significant. Meanwhile we analyzed whether the changes in expression of proteins involved in migration, such as RAC1, RHOA, RHOB, RHOC, and ROCK1 were consistent with the changes in the biological function, due to the overexpression and knockdown of DRAM2 in NSCLC cells. As results showed increased or decreased *DRAM2* expression enhanced or suppressed migration, respectively (Fig. 3).

### *DRAM2* promotes the proliferation of NSCLC cells

**Fig. 4:**
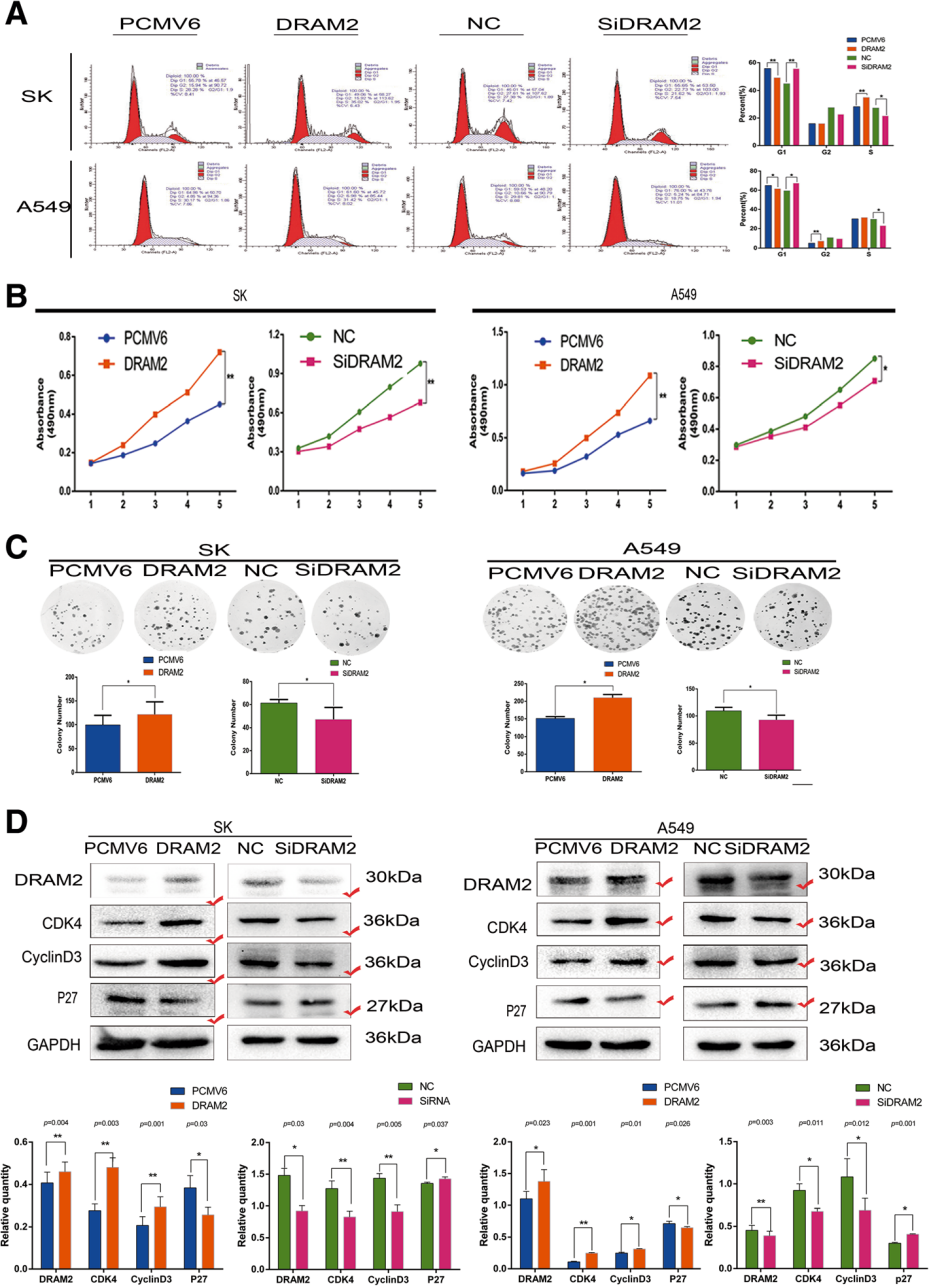
Expression of DRAM2 affected the proliferation of non-small cell lung cancer (NSCLC) cells. Upon DRAM2 overexpression and downregulation in SK and A549 cells, respectively, cell cycle analysis, MTT assay, and colony formation assay were performed. **a**. *p*-values of G1 phase of cell cycle analysis for A549 were: DRAM2 vs PCMV6, *p* = 0.022 and SiDRAM2 vs NC, *p* = 0.035; for SK, DRAM2 vs PCMV6 *p* = 0.003, SiDRAM2 vs NC, *p* = 0.016. *p*-values for differences in cells in G2 and S phase were also significant (data not shown); **b**. We compared MTT assay data on the fifth day, and calculated *p*-values for A549: DRAM2 vs PCMV6, *p* = 0.009; SiDRAM2 vs NC, *p* = 0.036; and for SK: DRAM2 vs PCMV6, *p* = 0.005; SiDRAM2 vs NC, *p* = 0.002; **c**. For colony formation assay in A549, DRAM2 vs PCMV6 *p* = 0.0164, and SiDRAM2 vs NC, *p* = 0.0462; for SK, DRAM2 vs PCMV6 *p* = 0.0465, and SiDRAM2 vs NC, *p* = 0.0310. *p < 0.05, **p < 0.01, ***p < 0.001 **d**. Effects of *DRAM2* levels on the expression of proteins associated with cell proliferation in transfected SK and A549 cells. The gray level of the proteins were detected, the corresponding *p* value were provided at the top of the graphs and those changed proteins were marked.*p < 0.05, **p < 0.01, ***p < 0.001

Next, we sought to determine whether the expression of *DRAM2* could affect cell proliferation of NSCLC, thus, cell cycle analysis, MTT assay, and colony formation were performed in A549 and SK cells after either overexpressing or downregulating *DRAM2,* and the differences in results for each assay were statistically significant (Fig. 4a-c)*.* Proteins involved in the cell cycle, including cyclin, cyclin-dependent kinases, and cell cycle inhibitory proteins were analyzed. Finally,we chose to focus on the changes in expression of proteins CDK4, cyclinD3, and p27 (Fig. 4d) and confirmed that *DRAM2* promoted proliferation of NSCLC cells.

### Expression of p53 and *DRAM2* are restrained by each other

**Fig. 5:**
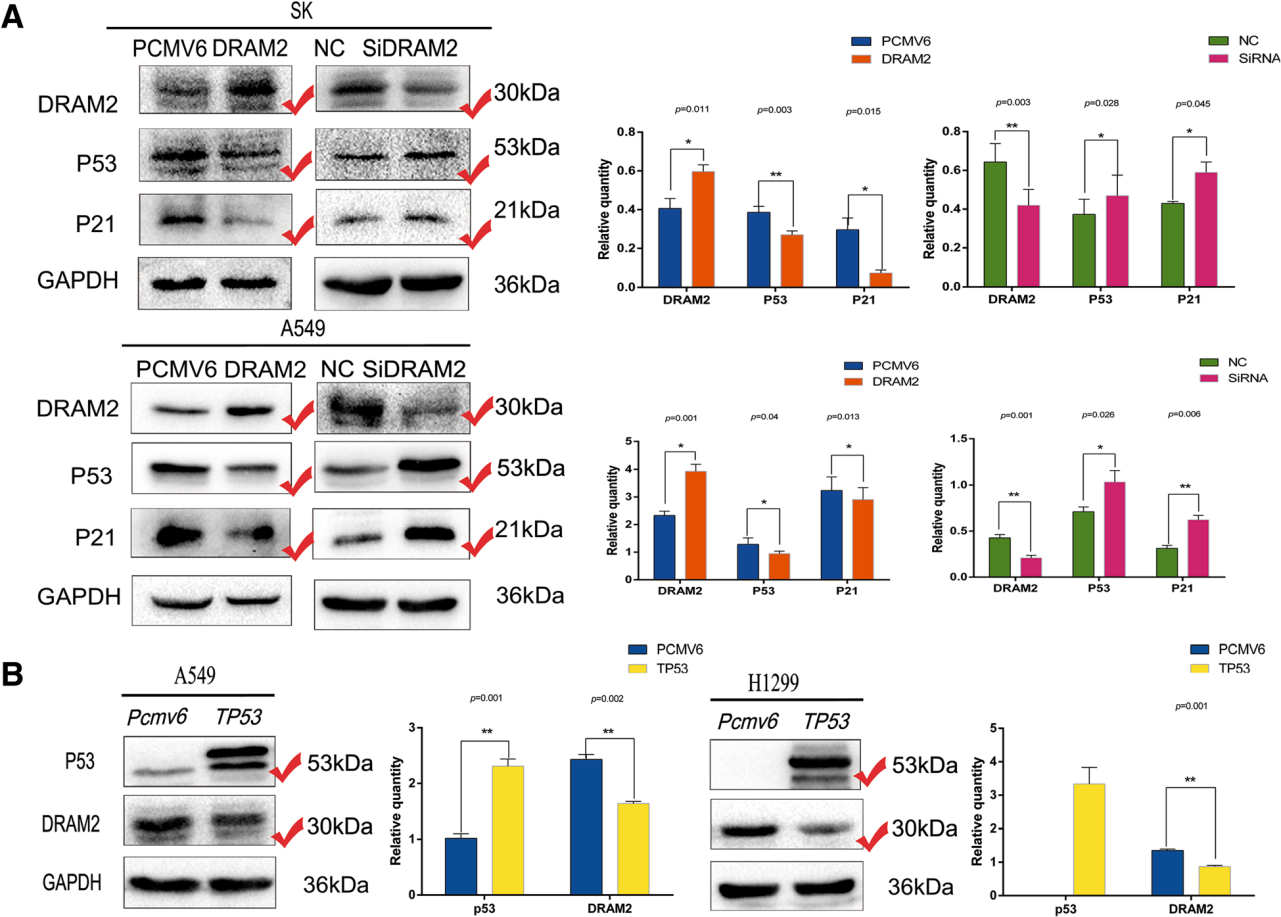
Expression of p53 and *DRAM2* were restrained by each other. **a** Protein p53 and its target, p21, were detected in the context of *DRAM2* overexpression and downregulation in SK and A549 cells, respectively. The gray level of the proteins were detected, the corresponding *p* value were provided at the top of the graphs and those changed proteins were marked.*p < 0.05, **p < 0.01, ***p < 0.001. **b** Protein DRAM2 was decreased after TP53 was transfected into A549 and H1299 cells. The gray level of the proteins were detected, the corresponding *p* value were provided at the top of the graphs and those changed proteins were marked.*p < 0.05, **p < 0.01, ***p < 0.001

Given that of the cell lines tested, *DRAM2* showed the lowest expression in H661 where endogenous p53 expression was more than 20 times higher than that of other cell lines (Fig. 2c), and the fact that *DRAM2* shares significant homology with *DRAM*, which can act either p53 dependently [26] or independently [27], it was necessary to explore the relationship between *DRAM2* and p53 in NSCLC. We first detected the expression of p53 and the p53 target gene, p21, while upregulating or downregulating *DRAM2* in A549 and SK cells, and found that the expression of p53 and p21 negatively affected by *DRAM2* expression(Fig. 5a). Next, we transfected TP53 to A549 and H1299 cells, then evaluated the expression of *DRAM2*, and found out that *DRAM2* was decreased when P53 was overexpressed(Fig. 5b). These results demonstrated that *DRAM2* and p53 interacted with each other and negatively affected each other’s expression.

### Expression level of p53 affected the biological behavior of oncogene *DRAM2*

**Fig. 6:**
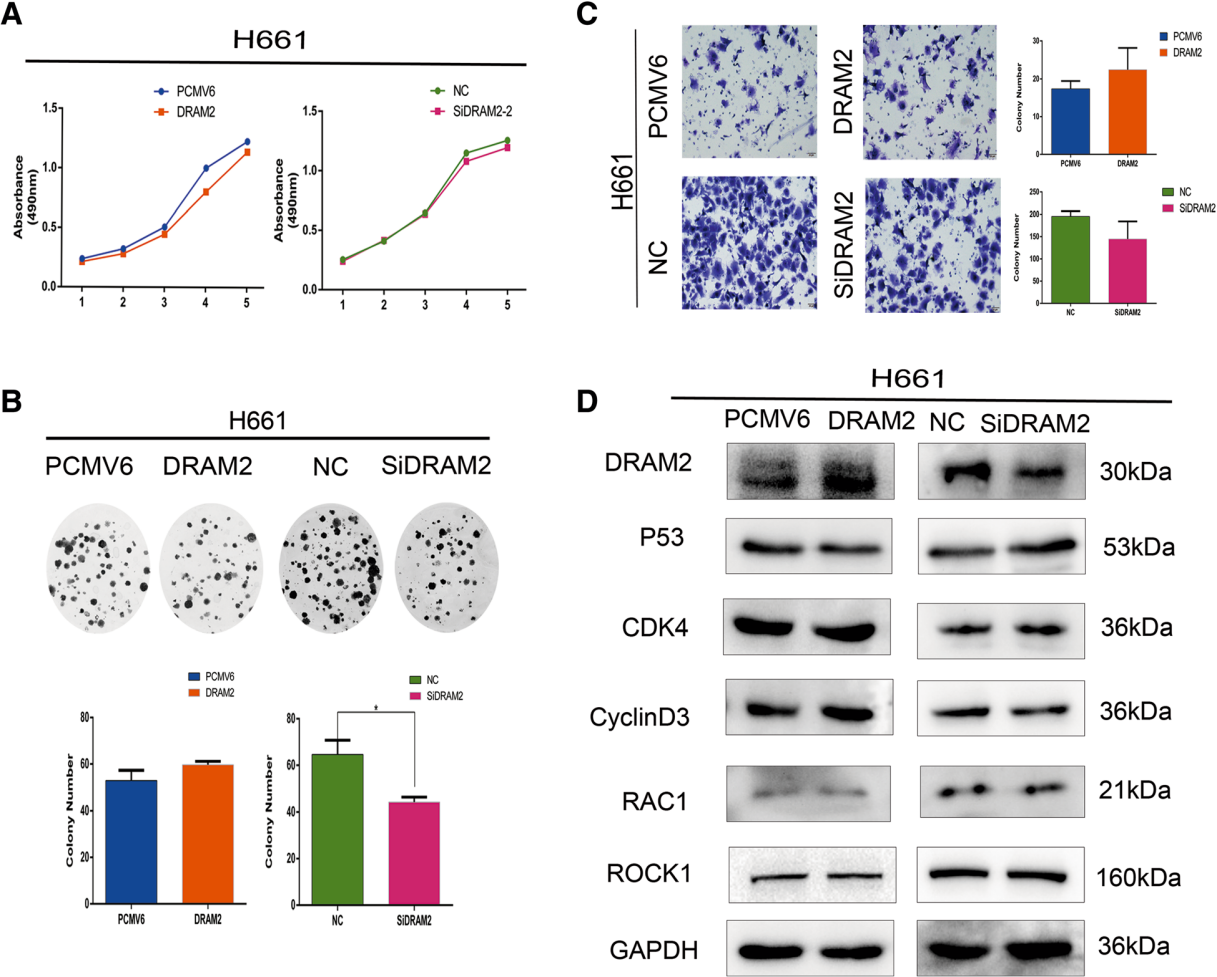
Overexpression of p53 restrained the effects of *DRAM2* on cell migration and proliferation. **a**. MTT assay; **b**. Colony formation assay; **c**. Transwell migration were performed in the context of *DRAM2* overexpression and downregulation in H661 cells. Only colony formation assay for both downregulated p53 and DRAM2 have statistical significance,*p* = 0.0482. **d**. Effects of *DRAM2* levels on the expression of proteins associated with cell proliferation, migration, and p53 in transfected H661 cells

**Fig. 7:**
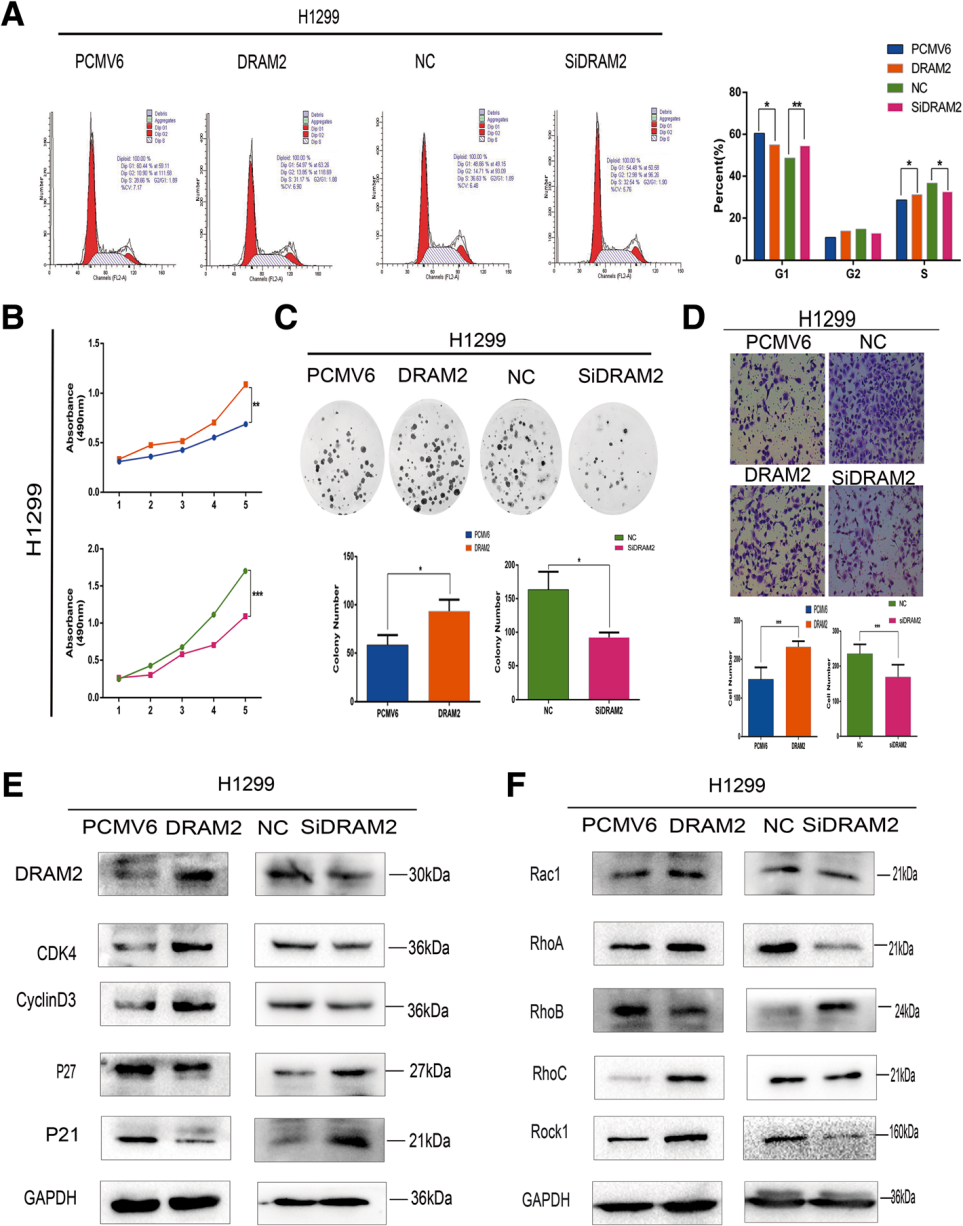
Absence of p53 did not affect the role of *DRAM2* in cell migration and proliferation. **a**. Cell cycle analysis; **b**. MTT assay; **c**. Colony formation assay; **d**. Transwell migration were performed in the context of *DRAM2* overexpression and downregulation in H1299 cells. *p*-values for differences in cells in G1 phase by cell cycle analysis for H1299 were *DRAM2* vs PCMV6, *p* = 0.019, and Si*DRAM2* vs NC, *p* = 0.009. *p-*values for differences in MTT assay data for the fifth day were *DRAM2* vs PCMV6, *p* = 0.014, and Si*DRAM2* vs NC, *p* = 0.001. The *p*-values for the colony formation assay were *DRAM2* vs PCMV6, *p* = 0.0334, and Si*DRAM2* vs NC, *p* = 0.0266. *p < 0.05, **p < 0.01, ***p < 0.001. **e**. Effects of *DRAM2* expression levels on the expression of proteins associated with cell proliferation in H1299 cells **f**. Effects of *DRAM2* expression levels on the expression of proteins associated with cell migration in H1299 cells

To investigate whether the expression of p53 is involved in functional activity of *DRAM2*, we chose the cell lines H661, which was p53 overexpressed, and H1299, which was p53 lacked, to downregulate and upregulate *DRAM2*, respectively, and performed transwell and MTT assays as well as colony formation and cell cycle analysis to observe the migration and proliferation changes. We compared the results with those in A549 and SK cells and found that p53 overexpression repressed the function of *DRAM2* in cell proliferation and migration (Fig. 6), which indicates that *DRAM2* may function as an oncogene via the p53-signaling pathway,and *DRAM2* still functioned in the absence of p53 suggests that *DRAM2* may choose another signaling pathway when p53-signaling pathway was blocked (Fig. 7). In sum, without p53 expression *DRAM2* served as an effective oncogene, and overexpression of p53 restrained the function of *DRAM2*.

## Discussion

We observed that DRAM2 was overexpressed in 170 out of 259 NSCLC tissues, and in 14 out of 20 NSCLC samples relative to that in normal tissues at the mRNA level, as well as in 12 out of 15 pairs of NSCLC tissue at the protein level. Moreover we discovered that the expression of DRAM2 related with TNM stage and lymph node metastasis. Then we found that the expression of *DRAM2* in NSCLC cell lines was higher than that in normal bronchial epithelial cell lines. Further, the biological behavior of A549 and SK cells with *DRAM2* either overexpressed or knocked down indicated that *DRAM2* contributes to the migration and proliferation of NSCLC, showing that *DRAM2* may be an oncogene in NSCLC. One limitation of our study was that we were unable to analyze the five-year survival rate of NSCLC patients given that the original samples were collected from 2014 to 2017, and insufficient time has lapsed for this data to be collected.

Given that the relationship between *DRAM2* and p53 has remained controversial, we felt it necessary to define their relationship as it pertains to NSCLC. Since we now have evidence that *DRAM2* represses the expression of p53, further studies should investigate how this occurs and which signal pathways *DRAM2* is involved in. Given the fact that when p53 was overexpressed, *DRAM2* did not act as a typical oncogene and appeared to serve as an oncogene when p53 was absent, we are left to conclude that *DRAM2* may work via repressing the p53 signaling pathway, but when p53 is absent, *DRAM2* may be forced to choose another pathway. Clearly, numerous questions remain, including whether *DRAM2* represses the expression of p53 through phosphorylation of p53, whether *DRAM2* is involved in p53 degradation, and whether *DRAM2* represses p53 directly or by targeting another protein to form complexes leading to change in p53 expression indirectly. In future studies, we also hope to clarify the relationship between *DRAM2,* autophagy [28], and apoptosis since *DRAM2* and *p53* are both strongly correlated with autophagy and apoptosis [20].

## Conclusion

In summary, in this article we showed that *DRAM2* is overexpressed in NSCLC and promotes migration and proliferation of NSCLC cells. Further, *DRAM2* repressed p53 expression and, conversely, p53 repressed *DRAM2* expression. Finally, similar to the negative relationship between *DRAM2* and p53 expression, the lower the expression of p53, the more successfully *DRAM2* functioned as an oncogene and vice versa. Elucidation of the mechanisms behind these findings is still needed. Furthering our knowledge of *DRAM2* will advance the possibility of this gene becoming a therapeutic target with clinical significance.

## Abbreviations

CDK4: Cyclin dependent kinase 4
DAPI: 4,6-diamino-2-phenyl indole
DMEM: Dulbecco Modified Eagle Medium
DMSO: Dimethyl sulfoxide
DRAM2: Damage-regulated autophagy modulator 2
ECL: Electrochemiluminescence
FACS: Fluorescence activated cell sorter
FBS: Fetal bovine serum
GAPDH: Glyceraldehyde 3-phosphate dehydrogenase
HBE: Human bronchial epithelioid cells
HRP: Horseradish peroxidase
MEM: Modified Eagle Medium
MTT: 3-(4, 5-dimethylthylthiazol-2yl-)-2, 5-diphenyl tetrazolium bromide
NC: Negative control
NSCLC: Non-small cell lung cancer
PBS: Phosphate-buffered saline
PVDF: Polyvinylidene fluoride
RAC1: Rac family small GTPase 1.
RHOA: Ras homolog family member A
RHOB: Ras homolog family member B
RHOC: Ras homolog family member C
ROCK1: Rho-associated protein kinase
SDS-PAGE: sodium dodecyl sulfate, sodium salt-polyacrylamide gel electrophoresis
siRNA: Small-interfering RNA
SPSS: Solutions Statistical Package for the Social Sciences
TBS: Tris-HCl buffer solution
TMEM77: Transmembrane protein77.
TNM: Tumor node metastases
